# Synthesis of Amphiphilic Block Copolymer and Its Application in Pigment-Based Ink

**DOI:** 10.3390/ma17020330

**Published:** 2024-01-09

**Authors:** Jingjing Yuan, Jinbao Xu

**Affiliations:** 1 Department of Art and Design, Taiyuan University, Taiyuan 237016, China; 2 School of Materials and Energy, Guangdong University of Technology, Guangzhou 510006, China

**Keywords:** amphiphilic block copolymer, PCL-*b*-PAA, inkjet ink, visual communication design

## Abstract

Amphiphilic block copolymers-based aqueous color inks show great potential in the field of visual communication design. However, the conventional step-by-step chemistry employed to synthesize the amphiphilic block copolymers is intricate, with low yield and high economic and environmental costs. In this work, we present a novel method for preparing an amphiphilic AB di-block copolymer of PCL-*b*-PAA by employing a combined polymerization strategy that involves both cationic ring-opening polymerization (ROP) of the *ε*-caprolactone monomer and the reversible addition–fragmentation chain-transfer (RAFT) polymerization on the acrylic acid monomer simultaneously. The corresponding polycaprolactone (PCL) and polyacrylic acid (PAA) serve as the hydrophobic and hydrophilic units, respectively. The effectiveness of the amphiphilic AB di-block copolymer as the polymeric pigment dispersant for water-based color inks is evaluated. The amphiphilic AB di-block copolymer of PCL-*b*-PAA exhibits a molecular weight of 1400 g mol^−1^, which is consistent with the theoretical value and suitable for polymeric dispersant application. The high surface excess (Γ_max_) of the PCL-*b*-PAA in water indicates a densely packed molecular morphology at the water/air interface. Additionally, micelles can be stably formed in the aqueous PCL-*b*-PAA solution at very low concentrations by demonstrating a low CMC value of 10^−4^ wt% and a micelle dimension of approximately 30 nm. The model ink dispersion is prepared using organic dyes (Disperse Yellow 232) and the amphiphilic block copolymer of PCL-*b*-PAA. The dispersion demonstrates near-Newtonian behavior, which is highly favorable for the application as inkjet ink. Furthermore, the ink dispersion displays a low viscosity, making it particularly suitable for visual communication design and printing purposes. Moreover, the ink dispersion demonstrates an unimodal distribution of the particle size, with an average diameter of approximately 500 nm. It retains exceptional stability of dispersion and even conducts a thermal aging treatment at 60 °C for 5 days. This work presents a facile and efficient synthetic strategy and molecular design of AB di-block copolymer-based dispersants for dye dispersions.

## 1. Introduction

In the recent decade, inkjet technology has emerged as a noteworthy and valuable method for visual communication design, finding application both in printing on paper and textiles, as well as in the precise fabrication of delicate electronic components, such as organic thin film transistors and organic light-emitting diodes [1,2]. In response to the growing concern for environmental issues, water-based inks have experienced a surge in their market share [3]. Notably, digital printing, specifically inkjet printing, has witnessed a steady rise in popularity owing to the increasing demand for on-demand production alongside mass production [4]. The quality of the printed materials heavily relies on the physical characteristics of the inks employed. In most cases, these inks exist as dispersions comprising dye particles and the aqueous liquid medium. Consequently, it is imperative to develop inks that are well-suited for specific deposition techniques and substrates in order to achieve the desired printing outcomes. This necessity arises due to the hydrophobic nature of typical colorants used in inks, namely organic pigments, which renders them incapable of dispersing in water [5]. Consequently, the use of dispersants is essential [6].

As one of the most important dispersants, polymeric dispersants find widespread usage in fulfilling diverse requirements, including the maintenance of ink stability, over a prolonged usage and temperature range and its fixation on the substrate after printing [7]. Given the hydrophobic nature of most organic pigments, the adsorption of the polymeric dispersants onto the dispersed pigments in an aqueous environment primarily relies on hydrophobic interactions [8]. Typically, a higher degree of hydrophobicity enhances the capacity loading for pigments, thereby leading to the improved dispersing performance of the dispersant [9]. Conversely, it is also desirable for the polymeric dispersant to possess good water solubility, thus facilitating the homogeneous ink formation as a dispersing agent [10]. Hence, in order to attain enduring ink dispersions, polymer-based dispersants must endeavor to achieve a judicious equilibrium between the hydrophobic and hydrophilic characteristics of the skeleton of the polymer [6,11]. This delicate balance facilitates robust and precise interactions with organic dyes while maintaining aqueous solubility. Keeping this notion in mind, considerable research efforts have been dedicated to the block copolymers, which consist of multiple functional segments and diverse compositions [12,13,14]. These copolymers serve as fundamental constituents in the advancement of polymer dispersant materials [15,16,17].

The amphiphilic block copolymers are a unique type of polymer with both hydrophobic and hydrophilic moieties. They have garnered significant research attention in the fields of polymer chemistry, supramolecular chemistry, biochemistry, etc. [18,19]. By characterizing the coexistence of distinctly opposing properties within a single molecule, these polymers have emerged as compelling surfactants [20]. The amphiphilic block copolymers inherently exhibit an extensive array of self-organized architectures that encompass a broad spectrum of structural arrangements, including micelles and vesicles, through particular molecular interactions, such as ionic or hydrophobic attractive forces [13]. This plays a pivotal role in shaping the overall structure of the micelles or the vesicles. Consequently, these unique properties make them highly suitable for application as ink dispersants. Historically, copolymers of poly(styrene-methacrylate) or poly(styrene-maleic acid) have enjoyed wide usage as amphiphilic polymers in the area of ink dispersions [21,22]. Nevertheless, recent advancements in this research field have predominantly focused on achieving effective dispersion of colorants in aqueous media by employing amphiphilic polymers. For example, Spinelli et al. conducted a comprehensive study to explore the potentials of amphiphilic polymers with diverse molecular topology, e.g., AB di-block copolymer, graft copolymer, and star-shaped copolymer, as polymeric dispersants for pigments [23]. His investigation delved into the intricate relationship between the chemical structures of amphiphilic polymers and their performance in achieving dispersion. Furthermore, Otake et al. reported on the dispersion effect of hydrophobic dyes using block copolymers comprising a hydrophilic 4-vinyl pyridine domain and a hydrophobic acrylate domain [24]. The study demonstrates that the dispersion performance is actually influenced by the characteristics of each segmental moiety of the block copolymers. These studies highlight the growing interest in utilizing amphiphilic polymers for the effective dispersion of colorants and the crucial role played by the specific structural attributes of these polymers in achieving desirable dispersion performance. However, the conventional step-by-step syntheses employed to synthesize amphiphilic block copolymers are intricate. As a result, the polymerization yield is low, and the associated economic and environmental costs are high. It is necessary to prepare amphiphilic block copolymers with low dispersity and well-defined chain structures via easy chemistry.

In inkjet ink technology, to prepare the ink dispersions with excellent dispersive stability and optimal viscoelastic properties, several necessities are essential [17]. In detail, the ink dispersion should exhibit high chemical and physical stability before inkjetting, allowing for stable storage without decomposition or sedimentation. During inkjetting, the ink droplets should follow a precise trajectory with high velocity, ensuring the distinct demarcation from the nozzle and inhibiting the formation of satellite droplets. The precise rheological characteristics and surface tension assume crucial roles in this phase. Finally, the ink droplets should exhibit good wettability, enabling rapid drying and guaranteeing the production of clear printed patterns after deposition onto the printing medium. The amphiphilic block copolymer-based dispersants can improve crucial factors such as the chemical stability, the trajectory linearity of the droplets, wettability, etc. To accomplish these objectives, the water-soluble amphiphilic polymers-based dispersant, should facilitate the ink solution’s high dispersion stability, appropriate rheological properties, and favorable surface characteristics. Preferably, amphiphilic block copolymers with increased hydrophobicity are preferred, capitalizing on their advantageous capacity for high pigment loading [25]. Due to the hydrophobic interactions between the hydrophobic segments of the polymer and the particles, the amphiphilic polymers experience adsorption onto the particle surfaces. Consequently, the dispersion of the particles in water is enhanced by means of the hydrophilic segments of the polymers [26]. In addition to the classical carboxylic acid groups, the inclusion of acrylic acid groups as water-soluble molecular constituents has also been extensively examined owing to their heightened acidity and hydrophilicity [27,28,29].

In this study, we present a straightforward synthetic approach to the fabrication of an AB di-block copolymer consisting of poly(*ε*-caprolactone)-block-poly(acrylic acid) (PCL-*b*-PAA). Our approach involves the combined polymerization of cationic ring-opening polymerization (ROP) and reversible addition–fragmentation chain-transfer (RAFT) polymerization chemistry. The copolymer’s hydrophobic component is the environmentally friendly ε-caprolactone (CL), while the hydrophilic segments are composed of acrylic acid (AA). Our primary objective revolves around investigating the effectiveness of the amphiphilic AB di-block copolymers as useful dispersant for dispersing pigments for visual communication design applications. To achieve this, we assess various properties such as critical micelle concentration, rheological behavior, Zeta potential, interfacial parameters, particle size distribution, etc., in aqueous media with a particular emphasis on aging tests at 60 °C for a duration of 5 days. Overall, our aim is to develop a practical polymeric dispersant for inkjet inks assisted visual communication design that demonstrates exceptional color reproducibility, admirable stability, and an extended lifespan in terms of jetting performance, using a facile, less costly, efficient and environmentally friendly synthetic approach.

## 2. Experiment

### 2.1. Materials

The raw materials for the synthesis of ROP initiator and RAFT agent of benzyl 2-hydroxyethyl carbonotrithioate (BSTSE, 99%), including 2-mercapto ethanol (99%), K_3_PO_4_ (98%), CS_2_ (99%), and benzyl bromide (99%), were all purchased from Aladdin chemical, China. The monomer of ε-caprolactone (99%) from Aladdin chemical was used after drying and distilled from CaH_2_. The monomer of acrylic acid was also purchased from Aladdin chemical and purified by flash chromatography with activated alumina before use. 2,2′-Azobis(2-methylpropionitrile) (AIBN, 98%) was also purchased from Aladdin chemical and used after recrystallized from methanol. Other chemicals, including acetone, N,N-dimethylformamide (DMF, 99%), tetrahydrofuran (THF, 99%), hexane (98%), triazabicyclodecene (TBD, 98%) and pyrene (99%) were all purchased from Aladdin chemical (Shanghai, China) and used according to the manufacturer’s instructions.

### 2.2. Synthesis of Benzyl 2-Hydroxyethyl Carbonotrithioate

**Scheme 1:**
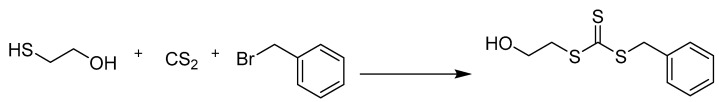
Synthetic approach for the difunctional ROP initiator and RAFT agent of BSTSE.

The synthetic approach for the difunctional ROP initiator and RAFT agent, BSTSE, is shown in Scheme 1. Briefly, 2-mercaptoethanol (1.00 g, 12.82 mmol) was added to a stirred suspension of K_3_PO_4_ (2.71 g, 12.82 mmol) in acetone (20 mL) and stirred for a duration of 10 min. Subsequently, CS_2_ (2.92 g, 38.46 mmol) was introduced, resulting in a vibrant yellow solution. After 10 minutes of stirring, benzyl bromide (2.19 g, 12.82 mmol) was swiftly added, resulting in instantaneous precipitation of KBr. The resulting suspension was stirred for an additional ten minutes and then filtered. The filter cake was washed with acetone twice (2 × 20 mL). After removing the solvent from the filtrate using rotary evaporator, a yellow viscous oil was received, which was subsequently purified by column chromatography on silica, utilizing a petroleum ether/ethyl acetate mixture as the eluent. (Yield: 90%).

### 2.3. Synthesis of Amphiphilic AB Di-Block Copolymer of PCL-b-PAA

**Scheme 2:**
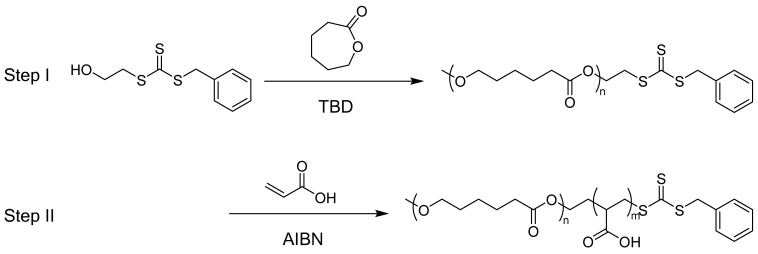
A combined ROP and RAFT reaction for the synthesis of the amphiphilic AB di-block copolymer of PCL-*b*-PAA.

Scheme 2 illustrates the combined synthetic approach of ROP and RAFT chemistry employed for preparation of the amphiphilic AB di-block copolymer PCL-*b*-PAA. Briefly, in a 100 mL-dried Schlenk flask, a solution containing *ε*-caprolactone (1.14 g, 10 mmol), BSTSE (0.58 g, 2.38 mmol, as both the ROP initiator and RAFT agent) and TBD (0.1 g) in 10 mL toluene was charged. The solution underwent degassing and backfilling with Ar. The solution was allowed to stir at ambient temperature for 24 h. The PCL precursor was collected by precipitation in hexane and dried at 60 °C for 24 h.

The AB di-block copolymer of PCL-*b*-PAA was prepared using the PCL precursor prepared above (1.72 g), acrylic acid (1.71 g, 24 mmol) and AIBN (0.039 g, 0.24 mmol, as a radical initiator) in 8 mL of DMF. The homogeneous solution was conducted with three freeze–pump–thaw cycles and allowed to react at 80 °C for a duration of 8 h. After the reaction, the solution was diluted with chloroform. The precipitation was made by adding hexane and collected. The crude product was washed with methanol and dried under vacuum at 60 °C to obtain the target amphiphilic AB di-block copolymer of PCL-*b*-PAA (Yield 82%).

### 2.4. Preparation of Ink Dispersion

The dispersing properties of the amphiphilic AB di-block copolymer PAA-*b*-PCL were assessed through the formulation of a pigment dispersion. Briefly, an aqueous polymer solution with a concentration of 15 wt% was prepared using the predetermined quantity of PCL-*b*-PAA and water. The polymer solution was then subjected to stirring and heatedat a temperature of 50 °C for a duration of 6 h, ensuring a fully dissolved and homogeneous state. Subsequently, a yellow disperse dye (Disperse Yellow 232) was added to the solution (1 wt%) [30]. Following this, zirconium dioxide (ZrO_2_) beads (size of 0.1 mm) were introduced alongside water into the resin vessel to serve as grinding media, thereby yielding a composite with an aggregate polymer concentration of 7.5 wt%. The mixture was stirred using a ball mill. After separating the beads via filtration, the filtrate was collected for measuring the rheological properties as well as the particle size distributions.

### 2.5. Characterizations

The proton NMR spectra (^1^H NMR) were acquired using a Bruker AV400 NMR spectrometer (Bruker Group, Zurich, Switzerland). The ^1^H NMR spectroscopy of BSTSE was performed utilizing CDCl_3_ as the solvent to verify its chemical structure, whereas the ^1^H NMR analysis of the AB di-block copolymer of PCL-*b*-PAA was conducted employing DMSO-*d6* as the solvent. In both cases, the residual solvent peaks (CDCl_3_: 7.26 ppm; DMSO-*d6*: 2.5 ppm) were utilized as the internal reference.

The molecular weight and polydispersity index of the AB di-block copolymer of PCL-*b*-PAA were determined by employing gel permeation chromatography (GPC) using a Shim-pack GPC-800D instrument from Shimazu (Trust Technology Co. Ltd., Kyoto, Japan) The calibration curve was achieved using polystyrene standards. NMP with LiBr (10 mM) was used as the eluent, with a flow rate of 1.0 mL/min and a temperature of 40 °C.

The critical micelle concentration of the amphiphilic AB diblock copolymer of PCL-*b*-PAA was measured utilizing a spectrofluorometer of FP-8050 from Jasco at a wavelength of 335 nm.

The particle size distribution of the micelles and the dye dispersion were determined using dynamic light scattering (DLS) using an ELSZ-1000 instrument from Otsuka Electronics (Otsuka electronics Co., Ltd., Osaka, Japan).

The determination of surface tension (γ) for the polymer solution was conducted using an automated surface tensiometer instrument of DY-300 from Kyowa (Kyowa Co., Ltd., Osaka, Japan), using the classical Wilhelmy plate method. In this method, a platinum plate was employed as the measuring instrument.

The measurement of Zeta potential (*ζ*) was carried out utilizing a Zetasizer Nano instrument of ZEN-3600 from Malvern (Malvern Panalytical, Malvern, UK).

The determination of viscosity for both the dispersion prior to and after aging was carried out using a modular compact rheometer instrument of MCR 300 from Anton Paar (Anton Paar GmbH, Graz, Austria).

Rheological measurements were conducted using a parallel-plate geometry with a plate diameter of 20 mm and a working distance of 100 μm. The rotational flow mode was used to evaluate viscosity dependence under shear rate sweeps from 0.01 to 1000 s^−1^ at 25 °C. The dye–polymer dispersions were analyzed at a solution concentration of 15 wt%.

The aging test was conducted by placing the ink dispersion in an oven at a temperature of 60 °C for a duration of 5 days.

## 3. Results and Discussion

### 3.1. Synthesis and Structural Characterization of BSTSE and Amphiphilic Block Copolymer

**Figure 1:**
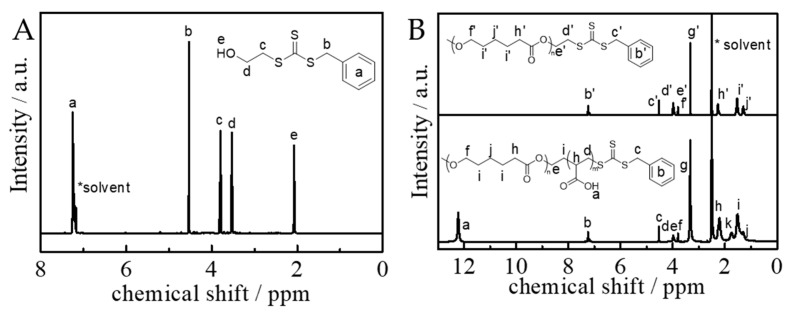
(**A**) ^1^H NMR spectrum of BSTSE. (**B**) ^1^H NMR spectrum of PCL precursor and the AB di-block copolymer of PCL-*b*-PAA (* represents solvent).

The synthesis of the amphiphilic AB di-block copolymer of PCL-*b*-PAA was achieved through a combined ROP and RAFT polymerization chemistry utilizing BSTSE as the ring-opening initiator and the chain transfer agent. The synthesis of the di-function ROP initiator and RAFT agent follows a procedure presented by O’Reilly et al. [31]. The chemical structure of the BSTSE is confirmed by ^1^H NMR, as shown in Figure 1A. The multiplet at the chemical shift of 7.39–7.23 ppm is assigned to the aromatic protons in benzene ring. The peaks at the chemical shift of 4.53, 3.80, 3.53, and 2.06 ppm are attributed to the C*H_2_*-Ph, S-C*H_2_*-CH_2_, S-CH_2_-C*H_2_*, and CH_2_O*H*, respectively. The ^1^H NMR result clearly confirms the successful synthesis of BSTSE, with the presence of the hydroxyl functionality and the carbonotrithioate group, which serve as the ring-opening initiator for ROP and the chain transfer agent for RAFT polymerization. Subsequently, the combined polymerization of ROP and RAFT chemistry was employed to prepare the amphiphilic AB di-block copolymer of PCL-*b*-PAA, in the presence of BSTSE. Once again, the successful synthesis of the material is confirmed by ^1^H NMR analysis, as shown in Figure 1B. The proton resonances are well assigned to the protons of the target block copolymer of PCL-*b*-PAA.

**Figure 2:**
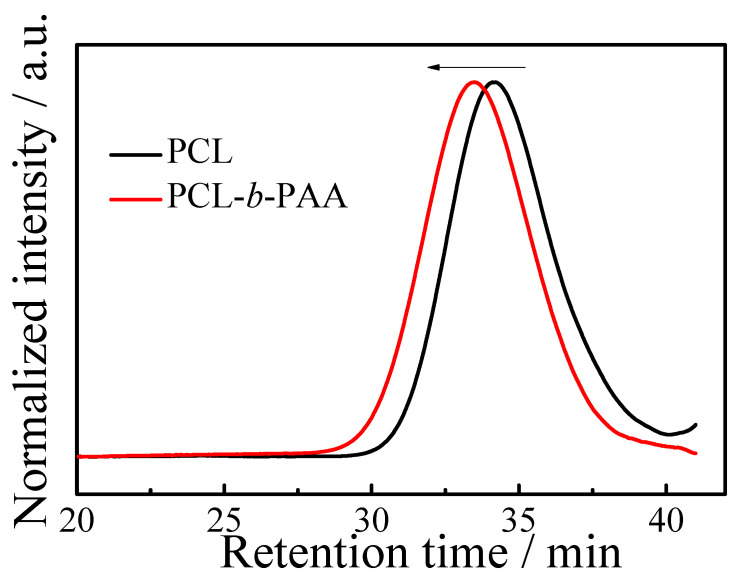
GPC traces of the PCL precursor and the amphiphilic AB di-block copolymer of PCL-*b*-PAA (The arrow indicates the increase of molecular weight).

In addition, the GPC trace of the amphiphilic block copolymers shown in Figure 2 reveals a typical unimodal peak, indicating the successful preparation of the block copolymer. The number average molecular weight (*M*_n_) and the weight average molecular weight (*M*_w_) are 1400 and 2400 g mol^−1^, respectively, aligning well with the theoretical value (*M*_n_ of 1200 g mol^−1^). The polydispersity index is 1.6, confirming the living polymerization mechanism. The molecular weight of the target polymer falls within an appropriate range, which facilitates the dispersion of dyes.

### 3.2. Aqueous Self-Assembly and Solution Behavior

**Figure 3:**
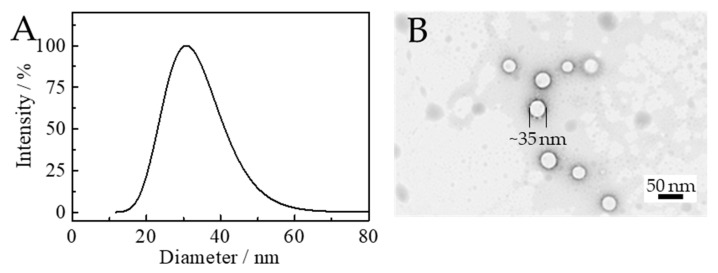
The micelle size distribution measurement of the amphiphilic AB di-block copolymer of PCL-*b*-PAA, using the 0.1 wt% aqueous polymer solution. (**A**) the DLS curve and (**B**) the TEM image of the micelles.

The solubility of the amphiphilic AB di-block copolymer in aqueous media is of utmost importance in relation to its application as a dispersant for inkjet ink. In order to prepare the aqueous solution, the as-prepared PCL-*b*-PAA was dissolved in water. The dimensions of the micelle particles were investigated through the utilization of dynamic light scattering (DLS) measurements and transmission electron microscopy (TEM) observation. The results, depicted in Figure 3, reveal that the PCL-*b*-PAA exhibits diminutive sizes, of approximately 30 nm, as shown in Figure 3A. The TEM image of the micelle provides visual confirmation, exhibiting an average diameter of ~35 nm (Figure 3B). This observation demonstrates that the amphiphilic block copolymer disperses effectively in water without the need for additional additives. It is anticipated that the block copolymer achieves molecular dispersion and self-assembly into polymer micelles within the aqueous milieu. This notion is further supported by the critical micellization concentration (CMC) measurement, as indicated below.

**Figure 4:**
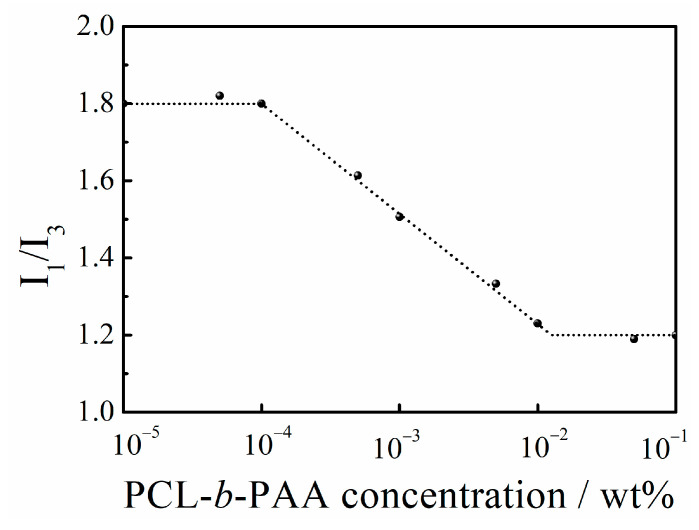
The I_1_/I_3_ value of pyrene as a function of the PCL-*b*-PAA concentration in polymer solution. The solution was prepared by adding 0.1 mL of 1 mM pyrene solution in ethanol and was irradiated by ultrasonication for 10 min.

To further investigate the behavior of the polymer dispersant in aqueous solutions, several diluted polymer solutions with various concentrations were examined with the addition of pyrene as a fluorescence probe. Typically, the amphiphilic polymer possesses a critical micellization concentration (CMC), where polymer micelles begin to form at concentrations surpassing the CMC [32,33]. The formation of micelles can be detected by monitoring the fluctuation in the fluorescence intensity ratio between the first and third vibronic emission bands of the introduced pyrene (denoted as I_1_/I_3_), of which the I_1_ is collected at 374 nm and the I_3_ is collected at 385 nm. The pyrene fluorescence fine structure is notably influenced by the solvent, thus the intensity ratio of I_1_/I_3_ exhibits high sensitivity to the solvent polarity [34]. Decreasing solvent polarity results in a reduction of I_1_/I_3_ values. When polymer micelles form in the aqueous solution, pyrene molecules infiltrate the hydrophobic core of the micelle, causing a decline in the polarity of the pyrene microenvironment and leading to a decrease in the I_1_/I_3_ value. As illustrated in Figure 4, the PCL-*b*-PAA demonstrates a clear decreasing trend in I_1_/I_3_ values, indicating the presence of the CMC. This finding aligns with the DLS results that identify the detection of polymer micelles in the AB di-block copolymer’s aqueous solution. Additionally, the block copolymer exhibits a significant decrease in I_1_/I_3_ value at a concentration of 10^−4^ wt%, which corresponds to the CMC value of PCL-*b*-PAA. These outcomes suggest that the behavior of the polymers in the solution affirms the fact that the polymers can dissolve in the aqueous phase while preserving their amphiphilic nature. Furthermore, it should be noted that the I_1_/I_3_ values of the block copolymer plateau at polymer concentrations exceed 0.01 wt%. The high polymer concentration beyond the CMC would result in an increased number of micelles in the solution, indicating a potential for accommodating a higher load of hydrophobic molecules such as pyrene or other dyes like dyes per unit weight of the block copolymer.

### 3.3. Rheology of Dye Dispersion with PCL-b-PAA

**Figure 5:**
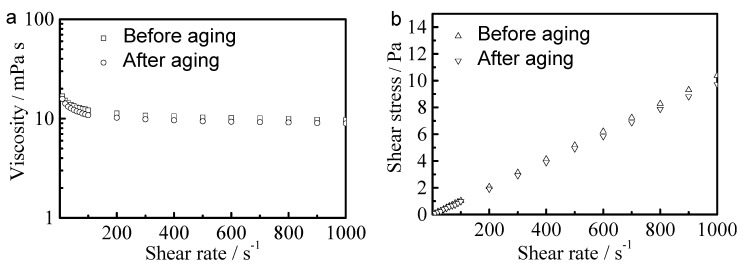
The viscosities (**a**) and the shear stress (**b**) of the dye dispersion as a function of shear rate. The test was performed before and after the aging test.

As previously discussed, the rheological characteristics of the dispersion play a crucial role in designing the optimal jetting conditions to achieve high-quality and consistent printed materials. To ensure stable inkjetting without clogging, it is essential to study the rheology of the dispersion inks, particularly the Newtonian characteristics at reduced viscosity, as this directly influences the efficacy of the nozzle, particularly under conditions of reduced shear rates [35]. Consequently, it is essential to assess viscosity encompassing a broad spectrum of shear rates, with the aim of ascertaining the optimal jetting conditions in accordance with the discerning needs of the clientele. Additionally, the viscosity and surface tension primarily govern the droplet formation. Inadequate viscosity leads to unstable droplet formation [36]. The ink dispersions in this study were created by blending the predetermined weight proportions of dye (Disperse Yellow 232), an aqueous solution of PCL-*b*-PAA, and ZrO_2_ beads (with a diameter of 0.1 mm). Following vigorous stirring using a planetary ball mill, the beads were extracted via the process of vacuum filtration, and, subsequently, the resultant filtrate was amassed for the purpose of studying its rheological characteristics and the DLS measurement of the particle size distribution. In Figure 5, the fluctuations in viscosities (5a) and the shear stress (5b) of the dispersion are plotted as a function of shear rates.

The dispersion containing dye and PCL-*b*-PAA exhibit typical near-Newtonian behavior, as depicted in Figure 5a,b. As depicted in Figure 5a, the dye dispersion demonstrates clear shear-thinning characteristics, reaching a maximum viscosity value of 16.8 mPa·s at a shear rate of 10 s^−1^. Previous studies on carboxymethyl cellulose solutions have attributed this shear-thinning behavior to polymer chain disentanglement or the heightened orientational arrangement of polymer coils [37]. In the instance of the present AB di-block copolymer of PCL-*b*-PAA, the interaction with dye particles has the potential to induce orientational alignment along the direction of flow. To guarantee the stability of inkjet printing, it is imperative to consider both the Newtonian properties and the orientational order of polymers within the ink solution as indispensable factors. Figure 5b further reveals that the dispersion containing PCL-*b*-PAA once again exhibits near-Newtonian behavior. The rheological characteristic observed can be ascribed to the intermolecular hydrogen bonding occurring among the carbonyl groups present in the acrylic acid constituents [38]. In order to evaluate the ink’s durability over an extended period within a limited period, an accelerated aging test was conducted. Both the particle size distribution and the rheological properties under temperatures surpassing the operational threshold were tested. The aging condition is implemented by aging at 60 °C for a long duration of 5 days. Rheological measurement of the dispersion after aging was carried out. After the aging test, there was a tiny decrease in both viscosity and shear stress for the ink dispersions. However, these changes are negligible, indicating the high chemical stability of the PCL-*b*-PAA dispersant and the overall stability of the dispersion.

### 3.4. Zeta Potential and Interfacial Parameter

The interfacial parameters play a vital role in comprehending the characteristics of surfactants at the interface between air and water. The determination of interfacial parameters offers valuable insights into the arrangement of the amphiphilic block copolymer at this interface, thereby enabling the evaluation of their effectiveness as dispersants. Therefore, by measuring the interfacial parameters of surface tension (*γ*) and surface excess (*Γ_max_*) vs. concentrations, it becomes possible to calculate the *Γ_max_* and the minimum occupied area per molecule (*A_min_*) using the Gibbs adsorption Equations (1) and (2), respectively. To simplify the model, the individual polymer chains are treated as large, single surfactant entities in this case.

(1) $$\Gamma_{max}=-\frac{1}{RT}\left[\frac{\partial \gamma }{\partial (\ln C)}\right]$$

(2) $$A_{min}=\frac{10^{18}}{N_{A}\zeta_{max}}$$

where *T* represents temperature (in K), *R* stands for the gas constant (8.314 J mol^−1^·K^−1^), and *N_A_* is the Avogadro’s number (6.02 × 10^23^ mol^−1^).

The calculated results are summarized in Table 1. The AB di-block copolymer of PCL-*b*-PAA exhibits a negative *ζ* potential value, implying the presence of negatively charged AA segments on the external surfaces of the aggregates [39]. The substantial magnitude of the *ζ* potential indicates the formation of non-ionic charged outer layers, leading to heightened electrostatic repulsion among the polymer chains [40]. This repulsive force contributes to a higher loading capacity for hydrophobic dyes and improves the storage stability of the system. Furthermore, the PCL-*b*-PAA amphiphilic block copolymer exhibits a low surface tension value, indicating its high surface activity. Consequently, stable layers are formed, thus alleviating the molecular connections among the H_2_O molecules. The minimum occupied area per molecule (*A_min_*) for PCL-*b*-PAA is determined to be 0.85 nm^2^ molecule^−1^. The relatively large *A_min_* value of the block copolymer can be attributed to the electrostatic repulsion effect caused the acrylic acid units, which leads to an expansion of their free volume [33]. This expansion allows the AB di-block copolymer of PCL-*b*-PAA to orient perpendicularly in the air/water interface.

### 3.5. Particle Size Distribution

**Figure 6:**
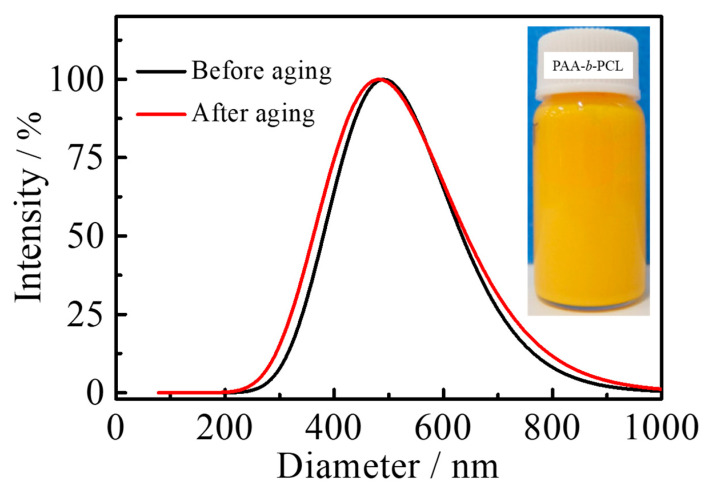
The particle size distribution of the ink dispersion containing the AB di-block copolymer of PCL-*b*-PAA.

Typically, the particle size of the ink dispersion has a substantial impact on the inkjetting performance and the quality of the ink. The particle size of the model ink with Disperse Yellow 232 and PCL-*b*-PAA is measured, to evaluate its excellence and resilience. Dynamic Light Scattering (DLS) was utilized to analyze the particle size of the ink dispersion, and the experimental results are depicted in Figure 6. The preparation of the ink dispersion has been clearly presented previously. The particle dimensions are chiefly governed by the viscosity of the polymer solution, due to the ball milling preparation process. In this study, the low viscosity of the polymeric dispersant solution enables efficient breakdown of the dye particles during ball milling, resulting in an average particle size distribution of approximately 500 nm. Moreover, the ink dispersions containing the AB di-block copolymer of PCL-*b*-PAA exhibit remarkable distribution stability even after aging for 5 days at 60 °C. The particle size distribution curve remains well-preserved following the aging test, indicating excellent dispersion stability. This notable stability in particle size distribution can be attributed to the existence of carboxylic acid functional groups in AA moieties, which effectively hinder excessive agglomeration of dye particles via electrostatic repulsion [41].

**Figure 7:**
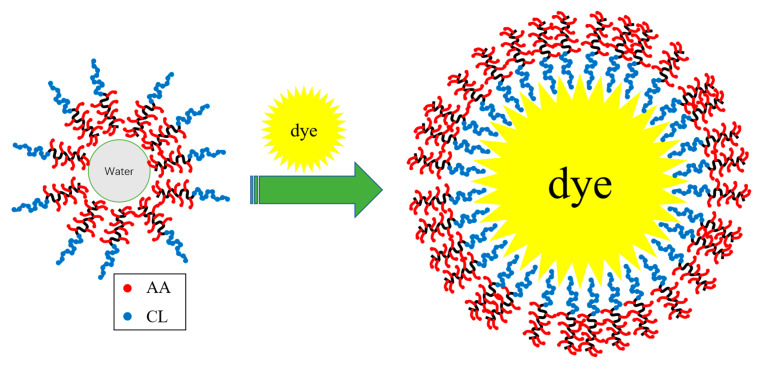
Possible aggregation behavior of the PCL-*b*-PAA in aqueous solution and with dye particle.

To date, the aggregation and sedimentation of several types of nanoparticles, including carbon nanotubes, iron (Fe) particles, and TiO_2_ nanoparticles are reported to arise from the physicochemical interactions [42,43,44,45,46]. A primary driving force behind the aggregation or sedimentation process is the diminution of electrostatic repulsion among the nanoparticles. The abhorrent interactions amidst the dye particles arise from the existence of ionic entities inherent within the adsorbed polymers [47]. In the current work, the AA polymeric layers are responsible for generating the repulsive electrostatic forces. The charged molecular entities undergo a conformational alteration, becoming integrated within the layer, thereby resulting in the concealment of the electrostatic force between particles. The amphiphilic AB di-block copolymer comprising PCL-*b*-PAA forms stable micelles in aqueous solutions, with the hydrophilic PAA component residing inside and the hydrophobic PCL segment on the exterior, as depicted in Figure 7. With the introduction of organic dye particles, the block copolymer forms enduring charged strata by means of robust adsorption onto the dye surface, facilitated by the concentrated hydrophobic functional groups. This process of adsorption culminates in the creation of steadfast inclusion complexes with the dye particles. Even under thermal aging conditions, the amphiphilic AB di-block copolymer of PCL-*b*-PAA demonstrates a high capability of maintaining its high stability under extreme conditions. The discourse on the durability of nanoparticle dispersions has extensively revolved around the concepts of electrostatic and steric stabilization. The pivotal factors that govern the agglomeration of nanoparticles, wherein the hydrophobic nature of the copolymer has been identified as a crucial determinant in attaining a stable dispersion of nanoparticles [48].

## 4. Conclusions

To summarize, a well-defined amphiphilic AB di-block copolymer, composed of acrylic acid and caprolactone moieties, is successfully synthesized for use as a dispersant in inkjet inks, catering to visual communication design application. The synthesis is achieved through a straightforward combined reaction involving ROP and RAFT chemistry. The obtained polymer solution exhibited a narrow size distribution with a peak size of approximately 30 nm. Furthermore, the polymer displayed a low CMC value of 0.01 wt% and exhibited a high loading capacity for hydrophobic molecules such as pyrene. This observation suggests that the polymers achieve a well-dissolved state at the molecular level in aqueous solutions. To assess the potential of the polymer for dye dispersion, important ink performance properties, namely viscosity and particle size distribution, were evaluated. The dispersion containing a yellow disperse dye (Disperse Yellow 232) and the AB di-block copolymer of PCL-*b*-PAA exhibited noticeable near-Newtonian behavior, with a low viscosity of 16.8 mPa·s at a shear rate of 10 s^−1^. The near-Newtonian properties and low viscosity of the PCL-*b*-PAA polymer enable effortless inkjetting and facilitate the production of high-quality printed objects. The low viscosity of the polymeric dispersant solution enables the efficient dispersing of the dye particles, resulting in an average particle size distribution of approximately 500 nm. Moreover, the ink dispersions containing the AB di-block copolymer of PCL-*b*-PAA exhibit remarkable distribution stability even after the aging test at 60 °C for 5 days. The experimental results indicate that the AB di-block copolymer of PCL-*b*-PAA effectively acts as a stable dispersant, preventing the sedimentation of dye particles.

## Figures and Tables

**Table 1:** Zeta (ζ) Potentials and interfacial parameters for the PCL-*b*-PAA aqueous polymer solution.

Sample	ζ Potential (mV) ^a^	γ (mN m^−2^) ^b^	*Γ_max_* × 10^6^ (mol m^−2^)	*A_min_* (nm^2^ molecule^−1^)
PCL-*b*-PAA	−14	48	2.05	0.85

^a^ The concentration of the aqueous amphiphilic block copolymer solution is 0.06 wt%. ^b^ The concentration of the aqueous amphiphilic block copolymer solution is 15 wt%.

## Data Availability

The data could be provided when it is required.
